# Germline and Somatic mutations in postmenopausal breast cancer patients

**DOI:** 10.6061/clinics/2021/e2837

**Published:** 2021-07-08

**Authors:** Tauana Rodrigues Nagy, Simone Maistro, Giselly Encinas, Maria Lucia Hirata Katayama, Glaucia Fernanda de Lima Pereira, Nelson Gaburo-Júnior, Lucas Augusto Moyses Franco, Ana Carolina Ribeiro Chaves de Gouvêa, Maria del Pilar Estevez Diz, Luiz Antonio Senna Leite, Maria Aparecida Azevedo Koike Folgueira

**Affiliations:** I Departamento de Radiologia e Oncologia, Instituto do Cancer do Estado de Sao Paulo (ICESP), Hospital das Clinicas HCFMUSP, Faculdade de Medicina, Universidade de Sao Paulo, Sao Paulo, SP, BR; II DB Molecular, Diagnostico do Brasil, Sao Paulo, SP, BR; III Departamento de Molestias Infecciosas e Parasitarias, Faculdade de Medicina FMUSP, Universidade de Sao Paulo, Sao Paulo, SP, BR

**Keywords:** Breast Cancer, Germline Mutation, Somatic Mutation, *BRCA1*, *BRCA2*, *PIK3CA*

## Abstract

**OBJECTIVES:**

In breast cancer (BC) patients, the frequency of germline *BRCA* mutations (g*BRCA*) may vary according to the ethnic background, age, and family history of cancer. Phosphatidylinositol-4,5-bisphosphate 3-kinase catalytic subunit alpha (*PIK3CA)* is the second most common somatic mutated gene in BC; however, the association of mutations in both genes with cancer has not been thoroughly investigated. Thus, our aims were to investigate g*BRCA* mutation frequency in a cohort of postmenopausal Brazilian BC patients and the association of g*BRCA1/BRCA2* and *PIK3CA* somatic mutations.

**METHODS:**

Forty-nine postmenopausal (>55 years) and forty-one young (≤35 years) BC patients were included in this study. The postmenopausal group included patients who reported a positive family history of cancer. For these patients, g*BRCA1/BRCA2* were sequenced using next-generation sequencing (NGS) or Sanger sequencing. Data for g*BRCA* in young patients were already available from a previous study. DNA from formalin-fixed, paraffin-embedded (FFPE) tumors was obtained from 27 postmenopausal and 41 young patients for analyzing exons 9 and 20 of *PIK3CA*. The association between g*BRCA1/BRCA2* and somatic mutations in *PIK3CA* was investigated.

**RESULTS:**

The overall frequency of g*BRCA1/BRCA2* among the 49 postmenopausal patients was 10.2%. The frequencies of somatic mutations in *PIK3CA* in the postmenopausal and young patients were 37% and 17%, respectively (ns). The most common *PIK3CA* mutation was found to be E454A. Nonsense and frameshift mutations, which may counteract the oncogenic potential of *PIK3CA* were also detected. Regardless of age, 25% of *BRCA1/BRCA2* mutation carriers and non-carriers , each, had *PIK3CA* somatic mutations.

**CONCLUSIONS:**

Data obtained indicate that *BRCA1/BRCA2* gene testing may be considered for postmenopausal patients with BC who have a family history of cancer. Although some of them are not considered pathogenic, somatic variants of *PIK3CA* are frequently observed in BC patients, especially in postmenopausal patients.

## BACKGROUND

Breast cancer affects women of all ages; however, the incidence of breast cancer increases with age, and the peak incidence occurs between 45-64 years (1). In addition, breast cancer is the most prevalent cancer in women aged 30-39 years (2). The main risk factors for breast cancer are a) age, b) positive family history of breast and ovarian cancer, and c) hormone exposure (3).

A positive family history is observed in approximately 10-20% of the breast cancer patients, but mutations in predisposing genes have been identified in <30% of these cases (4). *BRCA1/BRCA2*—both related to the homologous repair of DNA double-strand breaks—are the major breast/ovarian cancer susceptibility genes. Generally, women who harbor *BRCA1/BRCA2* mutations are more frequently diagnosed with breast cancer at an early age (≤40 years) or with ovarian cancer at any age. In addition, women who develop breast cancer at an older age and report a strong family history of breast/ovarian cancer mainly in close relatives—first, second, or third degree—may also be *BRCA1/BRCA2* mutation carriers (5). However, the majority of breast cancer cases are sporadic, *i.e.*, not related to genetic syndromes. In this case, somatic mutations accumulate over an individual’s lifetime, similar to an ‘evolutionary’ process, a phenomenon that makes age itself a risk factor for cancer (6). In this process, some cells acquire mutations that are advantageous from a tumoral perspective, which allows aberrant proliferation, invasion, and metastasis.

In breast cancer, somatic mutations in the *PIK3CA* gene are the most frequent, just after *TP53* (7). The *PIK3CA* gene encodes the p110 catalytic subunit of a heterodimeric lipid kinase called PI3K that is activated in response to various extracellular signals that are transduced through receptor tyrosine kinases. After activation, PI3K phosphorylates phosphatidylinositol-4,5-bisphosphate (PI-4,5-P2), generating phosphatidylinositol-3,4,5-trisphosphate (PIP3), which functions as a second messenger and recruits proteins that harbor pleckstrin homology (PH) domains (*e.g.*, AKT) (8). Mutations in the helical or kinase domain of *PIK3CA* resulted in the activation of the p110a kinase, with the subsequent downstream activation of mediators that culminates in cell proliferation, angiogenesis, and promotion of metastasis (9,10).

In breast cancer, an association between somatic mutations in *PIK3CA* and the positive expression of the estrogen receptor (ER) has been reported (11-14). However, the association between the frequency of somatic mutations in *PIK3CA* and age is unclear (15,16). Moreover, it seems likely that the frequency of somatic mutations in *PIK3CA* increases in ER-positive tumors in aging patients (7).

Thus, *BRCA1* and *BRCA2* are the most common germline mutated genes, while *PIK3CA* is the second most common somatic mutated gene in breast cancer patients; however, subtle frequency differences may be related to the age of onset of the disease. Carcinogenic mechanisms elicited by *BRCA1/BRCA2* loss of function and *PIK3CA* gain of function may be targeted for therapy. There is evidence that combination therapies targeting tumors harboring *BRCA* mutations—such as PARP inhibitors—with PI3K pathway inhibition therapies may exhibit synergy *in vivo* for the treatment of endogenous *BRCA1*-related breast cancer mouse model (17). However, it has been previously reported that the frequency of *PIK3CA* mutations may be different in breast cancer patients based on the presence of germline mutations in *BRCA1/BRCA2* (in both women and men) (18,19). Thus, our aim was to investigate the frequency of *BRCA* mutations in a cohort of postmenopausal Brazilian breast cancer patients, for whom scarce information is available. The secondary exploratory aim of this study was to evaluate the association of germline *BRCA1/BRCA2* mutations with somatic *PIK3CA* mutations in a cohort of young and postmenopausal patients with breast cancer.

## METHODS

### Patients

Patients were recruited at the Instituto do Câncer do Estado de São Paulo (ICESP), the cancer treatment branch of Hospital das Clínicas da Faculdade de Medicina da Universidade de São Paulo, the largest public hospital complex in Latin America, São Paulo, Brazil. This study was approved by the Institutional Ethics Committee (Comitê de Ética da Faculdade de Medicina da Universidade de São Paulo; protocol 397/11). All patients signed informed consent forms.

The inclusion criteria were 1) histopathological diagnosis of invasive breast carcinoma in patients aged <36 years or >54 years; 2) patients aged 55 years or older with at least one relative having first, second, or third degrees and diagnosed with breast, ovarian pancreatic, or prostate cancer; 3) triple-negative tumor and age ≤60 years. The expression of hormone receptor was classified as positive if at least 1% of the malignant cells were stained with antibodies against estrogen or progesterone receptor; HER2 positivity was defined as immunohistochemistry scores of 3(+) or 2(+), the latter, associated with fluorescence *in situ* hybridization (FISH)-amplification. HER2 immunohistochemistry and FISH were scored according to the ASCO/CAP guidelines (20). The Ki67 expression cut-off was set at >14% for a high proliferation index. The molecular subtypes were classified using previously established criteria (21).

Personal and familial cancer histories were collected through a structured questionnaire. Patients were also asked about their ancestry to obtain information about the country or continent where their parents and grandparents (at least) were born. A pedigree that reached up to third-degree relatives was designed. Clinical and pathological data were retrieved from hospital files.

In a previous study, 79 very young breast cancer patients (≤35 years) were evaluated for the presence of germline mutations in *BRCA1* and *BRCA2*, among whom, four harbored *BRCA1* mutations (c.66_67insA; c.211A>G; c.3331_3334delCAAG; c.5263_5264insC) and nine harbored *BRCA2* mutations (c.483T>A; c.1138_1138delA; c.2808_2811delACAA (n=2); c.3956_3959delATGA; c.6656C>G; c.6990_6994delTACCT; c.9154C>T; c.9382C>T) (22). For detecting *PIK3CA* mutations, tumor samples were available for 41 patients (among the 79 patients) and were included in the present analysis. Clinical data and tumor subtypes based on ER, PR, HER2, and Ki67 expression levels (as described above) are summarized in Table 4 (22). Six of these forty-one patients harbored *BRCA1* or *BRCA2* mutations.

### DNA Extraction from the Blood and Tumor Tissue

Genomic DNA from peripheral blood samples was extracted using the Illustra Blood Genomic Prep Mini Spin Kit (GE Healthcare Bio-Sciences, Pittsburgh, PA, USA), and from cancer cell-enriched areas from the formalin-fixed, paraffin-embedded (FFPE) tumor samples using the QIAamp^®^ DNA FFPE Tissue (Qiagen, Valencia, CA, USA), as per the manufacturer's protocol.

DNA concentration and purity were determined using a NanoDrop 1000 Spectrophotometer (Thermo Fisher Scientific, Massachusetts, USA), and the absorbance_260/280_ ratio varied from 1.42 to 2.2. DNA concentration from samples analyzed using next-generation sequencing (NGS) was also evaluated using a Qubit^®^ dsDNA BR Assay kit on a Qubit^®^ 3.0 Fluorometer (Invitrogen, Carlsbad, California, USA).

### Analysis of Germline Mutations in *BRCA1*/*BRCA2*

The entire coding regions of *BRCA1* and *BRCA2*, including exon-intron boundaries, were sequenced by NGS using the Ion Torrent Personal Genome Machine (PGM) platform (n=38) or by Sanger sequencing (n=11), to determine the presence of germline mutations.

### Next-Generation Sequencing

*BRCA1 and BRCA2* were sequenced using the Ion AmpliSeq™ *BRCA1* and *BRCA2* Panel (Life Technologies, Carlsbad, CA, USA) consisting of three primer pools, covering the target regions in 167 amplicons that target the entire coding region, including 10-20 bp of non-coding sequences, flanking the 5’ and 3’ ends of each exon, for both genes. Libraries containing the PCR product were sequenced on a 314 v2 Ion Chip, which allows the simultaneous analysis of 12 samples per chip on a PGM sequencer (Ion Torrent™), and the Ion PGM Sequencing 200 Kit version 2 (Life Technologies, Carlsbad, CA, USA). Data analysis was performed using the Ion Reporter™ Server System (Thermo Fisher Scientific, Massachusetts, USA). Sequence data were also visually evaluated using the Integrative Genomics Viewer (IGV). Amplicons with coverage less than 30x, pathogenic variants, and new variants were confirmed by PCR followed by conventional bidirectional Sanger sequencing. Full details of the methods are provided in the Appendix.

### PCR and Sanger Sequencing

All coding regions, including the intron-exon boundaries of *BRCA1* (NM_7294.3) and *BRCA2* (NM_000059.3) were amplified by PCR. Primers and conditions are described in the Appendix. The amplicons were purified (Illustra™ ExoStar™ 1-Step-GE Healthcare Bio-Sciences, Pittsburgh, PA, USA) and were sequenced using the BigDye^TM^ Terminator v3.1 Cycle Sequencing kit (Applied Biosystems^TM^, Foster City, California, USA), as described previously (22). Following purification, samples were analyzed on a 3500 Genetic Analyzer or ABI 3730 DNA Analyzer (Applied Biosystems™, Foster City, California, USA) in both forward and reverse directions (Appendix). The results were analyzed using Mutation Surveyor DNA Variant Analysis Software (v3.30, SoftGenetics LLC). All pathogenic mutations were confirmed using Sanger sequencing.

### Analysis of Copy Number Variation in *BRCA1* and *BRCA2*

For the analysis of large deletions and duplications—that would have provided comprehensive information regarding germline mutations—patient DNA was subjected to *BRCA1* and *BRCA2* multiplex ligation-dependent probe amplification (MLPA) analysis (*BRCA1*: SALSA^®^ MLPA^®^ P002 and P087 Probemix; *BRCA2*: SALSA^®^ MLPA^®^ P045 *BRCA2/CHEK2* Probemix; MRC-Holland, Amsterdam, The Netherlands), as per the manufacturer's protocols (Appendix), as described previously (22,23).

### Mutation Nomenclature and Classification

*BRCA1* and *BRCA2* variants were named according to the Human Genome Variation Society (HGVS) nomenclature (24) and were searched in publicly accessible databases, *i.e.*, BRCA Share™, BRCA Exchange, BRCA Mutation Database, and ClinVar. The search was performed in 2020 (between January and June). In *silico* analyzes were performed using the following prediction tools: Polymorphism Phenotyping (PolyPhen; v2.2.2), Sorting Intolerant From Tolerant (SIFT; v1.0.3), Align-GVGD, Protein Variation Effect Analyzer (Provean; v1.1), and Human Splicing Finder to analyze variants of unknown clinical significance. Minor allele frequency (MAF) was checked on the 1000 Genomes Project database, Exome Aggregation Consortium (ExAC), Global MAF dbSNP, Exome Variant Server, NHLBI GO Exome Sequencing Project (ESP), Genome Aggregation Database (gnomAD), Trans-Omics for Precision Medicine (TOPMed), and Brazilian genomic variants (ABraOM). More details are provided in the Appendix.

The variants were then classified as pathogenic, likely pathogenic, benign, likely benign, and variant of uncertain significance (VUS) based on the recommendations of the American College of Medical Genetics and Genomics (25). VUS for *BRCA* was also checked for co-occurrence with known pathogenic mutations in the same patient. For some variants, we considered that consensus information in ≥2 databases was strong enough to classify them as benign or VUS.

### Analysis of Somatic Mutations in *PIK3CA*

**Figure 1:**
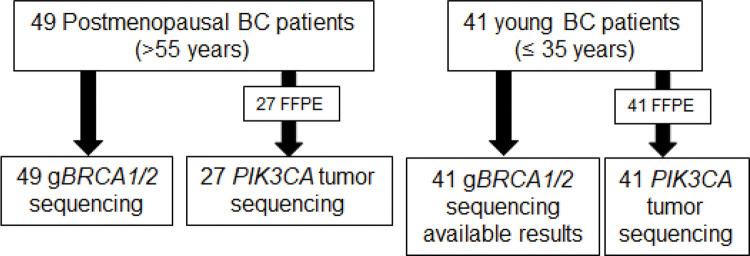
The flowchart summarizes the samples used for each analysis.

Among the 49 postmenopausal patients, 27 FFPE tumor samples were available for analysis. Tumor samples from another 22 patients were not available because they had been operated on at another service. Tumor samples from all 41 young patients were used for further analysis (Figure 1).

*PIK3CA* (NM_006218.2) exons 9 (helical domain) and 20 (kinase domain), which are the regions with the highest mutation frequency (26), were amplified by PCR and were analyzed by Sanger sequencing. Primer sets were designed using software Primer3 (http://bioinfo.ut.ee/primer3/). To avoid non-specific product formation, BLAST (http://www.ncbi.nlm.nih.gov/blast) and BLAT (https://genome.ucsc.edu/cgi-bin/hgBlat) were performed. Primers and conditions are described in the Appendix.

### Statistical Analysis and Sample Size Calculation

To detect the frequency of germline *BRCA* mutations in postmenopausal breast cancer patients (varying from 2% to 17%), a sample size of 50 was estimated (27,28). For analyzing the frequency of *PIK3CA* mutations in young and postmenopausal patients; this was a convenient sample size, because only 55% of tumor samples were available for the latter. Assuming that the frequency of *PIK3CA* mutations in young and postmenopausal patients was 7% and 35%, respectively (7) and the correlation of two postmenopausal patients for every three young patients, the estimated sample size to detect a difference with 0.05 one-sided significance level and 80% power would be 31 young and 21 postmenopausal patients.

Pearson’s chi-square test was used to evaluate the association between variables, and a two-sided significance level of 0.05 was considered.

## RESULTS

### Patients

Forty-nine elderly women aged ≥55 years who were diagnosed with invasive ductal breast carcinoma were included between May 2014 and May 2015 and evaluated for the presence of germline mutations in *BRCA*. FFPE tumor samples of 27 patients were analyzed for the presence of somatic mutations in *PIK3CA*. The median ages at the time of diagnosis and enrollment in the study were 61 years (55-80 years) and 64 years (56-87 years), respectively. The majority of the patients had Nottingham histological grade II tumors (63.3%) and clinical stage I/II tumors (67.4%). With respect to the tumor subtype, most tumors were luminal B (44.9%) or luminal A (22.4%), followed by HER2^+^ and triple-negative tumors (10.2% each) (Table 1; Additional Table 1). Most patients (95.9%)—except for two patients (one with a triple-negative tumor and age ≤60 years)—reported a positive family history of breast, ovarian, pancreatic, or prostate cancers. A large proportion of the patients (69.4%) reported at least one affected first-degree family member with breast and/or ovarian cancer. Most women were born in the Southeast (67.3%)—followed by the Northeast (18.4%)—regions of Brazil. With respect to ancestry, 28.6% of the patients reported Brazilian and European ancestries, 26.5% reported only Brazilian ancestry, and 18.4% and 8.4% reported European-only or Asian ancestry, respectively (Table 1).

Another 41 young patients, aged ≤35 years, had their tumor samples evaluated for the presence of somatic mutations in *PIK3CA*. This is a subgroup of patients whose clinical data, as well as germline *BRCA1* and *BRCA2* sequencing results had already been reported in a previous study (22). The cohort of patients now reported comprehends those young patients who had FFPE tumor samples available for *PIK3CA* analysis. The median age at the time of diagnosis was 32 years (range, 23-35 years). Most patients presented tumors with histological grade II (43.9%) or III (48.8%), and disease clinical stage I/II (65.7%). Luminal B (46.3%) was the most frequent tumor subtype, followed by triple-negative (24.4%) and HER2 (+) (12.2%) tumors (Table 4). Among these patients, 14.6% and 12.2% reported first-or second-degree relatives diagnosed with breast and/or ovarian cancer, respectively, while 39% reported a negative family history of breast and/or ovarian cancer, and 24.4% were not able to describe their family history. Six out of the forty-one patients harbored pathogenic mutations (14.6%) in *BRCA1* or *BRCA2*, as previously reported (22).

### Germline Mutations in *BRCA1* and *BRCA2* in Postmenopausal Patients

Among 49 postmenopausal unrelated women, 5 (10.2%) were identified to harbor mutations of clinical significance, 3 in *BRCA1* and 2 in *BRCA2* (Table 2; Additional Tables 2-3). All five *BRCA* mutations were identified among 47 patients who reported a positive family history of breast, ovarian, prostate, and pancreatic cancers in close relatives (10.6%), including four mutations detected among 34 patients reporting first-degree relatives affected by these types of cancer (11.76%) (Table 1).

Mutations in *BRCA1* comprised one splice-site variant (c.5074+2T>C, in exon 17), one missense mutation (c.5123C>A), and one *BRCA1* rearrangement generating a large deletion encompassing exons 1-19. The two pathogenic mutations in *BRCA2* included one missense variant (c.2T>G) and one nonsense variant (c.5645C>A) (Table 2). The presence of *CHEK2* c.1100delC mutation was investigated in 47 out of the 49 patients; however, no mutations were detected. 

Eight VUS were detected, five in *BRCA1* and three in *BRCA2*. Among the VUS, four distinct missense variants were identified, two in each gene (*BRCA1*: c.3305A>G and c.3752G>A; *BRCA2*: c.3371A>G and c.8942A>G), among which *BRCA2* c.3371A>G was predicted to be deleterious by at least three out of four mutation function prediction models (SIFT, Polyphen-2, Align-GVGD, or Provean) (Table 3). The remaining VUS were located in the intronic regions, at least 36 nucleotides away from the intron-exon boundary.

### Presence of Somatic Mutations in *PIK3CA* in Postmenopausal and Young Patients

Tumor sequencing was performed on samples from 27 elderly patients to identify *PIK3CA* mutations. Fourteen tumors (51.8%) were found to harbor mutations in exons 9 or 20; however, only ten (37%) harbored meaningful deleterious or possibly deleterious variants (pathogenic in at least one out of four function prediction tests). Recurrent mutations were E545A (observed in four samples) and H1047L (in the other two samples). Among these 27 elderly patients, two were *BRCA1* mutation carriers, both of whom harbored somatic pathogenic (E545A) or possibly pathogenic *PIK3CA* mutations (Additional Table 4).

Another three tumors (11.1%), all luminal A subtypes, harbored synonymous variants (in one case, associated with an intronic variant) (sample 39). In addition, tumors from another six patients harbored multiple *PIK3CA* variants; however, two tumors harbored (samples 26 and 39) a combination of non-pathogenic variants represented by missense non-pathogenic and nonsense variants (sample 26) or a combination of a deep intronic and two synonymous variants (sample 39). In the third tumor, *PIK3CA* double mutation (sample 47) (S541P and E1037V) was considered pathogenic in at least three function prediction tests, even though none of them were located in a hotspot. In the fourth and fifth tumors (samples 36 and 46), the contribution of the mutations were difficult to define because the *PIK3CA* pathogenic missense variant (E545A) was accompanied by a frameshift (FS) mutation (S553FS). If it occurs in cis, FS S553FS might counteract the oncogenic potential of E545A. The sixth tumor (sample 8) harbored a pathogenic hotspot (H1047L) and a synonymous variant (Additional Table 4).

In a cohort of young patients, *PIK3CA* variants were observed in 12 tumors, including synonymous variants—detected in two tumors (one luminal B, sample 484, and one HER2^+^ sample 503)—and missense non-pathogenic variants detected in another two samples (samples 455 and 478). In addition, a nonsense variant, W552* was detected in a luminal A tumor (sample 468). Hence, pathogenic or possibly pathogenic *PIK3CA* mutations were detected in seven out of forty-one young patients (17.1%) (Additional Table 5).

Among the young patients, E545A was the most frequent mutation (detected in three different samples, one luminal B and two triple-negative tumors). In one of these triple-negative tumors, E545A occurred concomitantly with N1068T, another pathogenic variant. The variant P539S, considered pathogenic in the prediction models, was detected in two luminal B samples, in one of these cases, in combination with R555K, which is also a pathogenic variant.

We then compared frequency of pathogenic *PIK3CA* mutation in tumors from postmenopausal and young patients (37% *vs.* 17%); however, we could not find a significant difference (Table 4). Using our data with a sample size of 27 postmenopausal and 41 young women and the reported frequency of *PIK3CA* mutation, the power to detect a difference with a one-sided significance level of 0.05% was 58.51%.

The frequency of *PIK3CA* is enriched in ER-positive tumors, and in a previous study we detected a trend toward a higher frequency of *PIK3CA* mutations in ER-positive tumor from elderly women compared to that observed in younger women (7). Upon considering the characteristics of the patients in the present series, we observed differences between the two groups, reflecting a higher proportion of luminal tumors in postmenopausal women. We then analyzed the frequency of *PIK3CA* mutations in luminal tumors and observed that eight out of the twenty-four samples (33.3%) from postmenopausal patients and five out of the twenty-five samples (20%) from young patients harbored pathogenic *PIK3CA* mutations (Table 4; *p*=0.291). A future meta-analysis including more recent data may help to clarify this aspect.

We next considered a total of 68 patients, postmenopausal as well as young, who were tested for the presence of germline mutations in *BRCA1/BRCA2* and somatic mutations in *PIK3CA*. Upon simultaneously considering patients from both age groups, two out of eight germline *BRCA1/BRCA2* mutant carriers (25%) were also found to harbor somatic mutations in *PIK3CA*. Among the 60 patients who were *BRCA1 and BRCA2* wild type, 15 manifested tumors harboring *PIK3CA* mutations (25%).

## DISCUSSION

In this cohort of postmenopausal breast cancer patients, 10.2% harbored pathogenic germline *BRCA1/BRCA2* variants; 11.7% of these patients had at least one family member who was affected with breast, ovarian, prostate, or pancreatic cancer.

Age at the onset of breast cancer and a family history of breast and ovarian cancer are important factors associated with the frequency of germline *BRCA* mutations (29). For elderly patients who were not selected for a family history of cancer, the frequency of *BRCA* mutations tended to be relatively low. Accordingly, a recent nested case-control study conducted in the USA revealed that only 1.18% of the unselected postmenopausal breast cancer patients were *BRCA1/BRCA2* mutation carriers (27). In a large cohort comprising 1554 Brazilian breast cancer patients referred for genetic testing at a single clinical diagnostic laboratory in Brazil, 9.84% were found to be *BRCA1* or *BRCA2* mutation carriers independent of age (30). Higher *BRCA* mutation frequencies (varying from 15% to 22%) have been reported among young Brazilian breast cancer patients with ages up to 35 years (22,31,30). However, specifically for postmenopausal Brazilian patients with breast cancer, little data are available. Our study indicates that 10.6% of the breast cancer patients with at least one close relative affected by the disease (until third degree) harbor germline *BRCA1/BRCA2* mutations. A previous study evaluated 39 breast cancer patients aged more than 50 years, among whom 17.9% were *BRCA* mutation carriers (32). These latter patients reported a strong family history based on the early age of cancer onset or multiple relatives with breast cancer and/or ovarian cancer at any age, which may explain the higher *BRCA* mutation frequency.

An important issue to take into consideration is the cost-effectiveness of the diagnostic program for germline mutations in *BRCA1/BRCA2* genes and preventative strategies for relatives of patients diagnosed with the mutation. In the scenario of Brazilian ovarian cancer patients, for whom *BRCA1/BRCA2* mutation frequency is 20%, performing genetic testing and adopting prophylactic measures for family members was considered a cost-effective measure (33). In a more inclusive model, *BRCA* testing may be offered to women of the general population to avoid missing mutation carriers, owing to test indications based on clinical criteria and family history. In this context, population-based *BRCA* testing was estimated to be cost-effective for the Brazilian population and to prevent a large number of breast and ovarian cancer cases (34). Although direct studies for postmenopausal Brazilian breast cancer patients are necessary, the previous two studies might suggest that genetic testing may be valuable for these women in the context of a positive family history.

The variants detected in the present study were not among the most frequent mutations in *BRCA1* and *BRCA2* in Brazilian patients with breast cancer. Variants *BRCA1* c.5074+2T>C, *BRCA1* c.5123C>A, and *BRCA2* c.2T>G respectively represent 2.2%, 0.5%, and 1.2% of the *BRCA1/BRCA2* mutations previously reported (28).

The other two *BRCA* mutations, *BRCA1* large rearrangement (del exons 1-19) and *BRCA2* c.5645C>A, have not been previously reported in the Brazilian population. The variant, *BRCA2* c.5645C>A has been reported in breast cancer patients from Japan, China, and the Czech Republic (35,36,37), and in prostate cancer patients (38). Interestingly, our patient who harbored this variant was also born in Japan.

Somatic mutations in *PIK3CA* gene are the second most common mutations in breast cancer, just after *TP53* (7). The *PIK3CA* mutation hotspots were clustered in exon 9 in nucleotides corresponding to codons E542K and E545K (helical domain) and in exon 20 in nucleotides corresponding to codon H1047R (kinase domain) (39,40).

In the present series, the most frequent mutation in *PIK3CA* in tumors from both postmenopausal and young patients was E545A, a variant with intermediate oncogenic potency, located in the helical domain (39). In agreement with our data, studies on breast cancer patients from Singapore and Peru have also found E545A to be the most frequent *PIK3CA* variant in tumor samples (41,42). Nevertheless, a method was developed to specifically enhance the detection of E454A (43). In contrast, data from another cohort of Brazilian patients with sporadic breast cancer have reported that the most frequent *PIK3CA* hotspot mutations were E542K, E545K, and H1047R (13).

The second most commonly found mutations in elderly patients were H1047L and S553FS. H1047L is located in the kinase domain and is associated with high oncogenic potential (39). Further, the frameshift mutation S553FS might counteract the proto-oncogene potential of *PIK3CA*. In addition, nonsense mutations were detected in tumors from both elderly and young patients, which might also neutralize the proto-oncogenic activity of *PIK3CA*. However, another study has specified that nonsense mutations in *PIK3CA* are not frequently encountered (44).

Six tumors were found to harbor double or triple *PIK3CA* variants (four from elderly patients and two from young patients). It has been previously shown that approximately 13% of all the *PIK3CA* mutations correspond to multiple variants occurring in the same tumor. It has also been reported that most double mutations occur in *cis* and induce the activation of the downstream PI3K pathway (compared to single-hotspot mutants) (40). However, in the present study, among the four tumors in elderly patients harboring double or triple variants, only one might be deleterious, involving a combination of S541P and E1037V. In the other three tumors, concomitant variants included nonsense, frameshift, synonymous, and intronic variants, in addition to missense variants with pathogenic or non-pathogenic potential. The determination of whether these variants were in *cis* might have helped to determine the oncogenic potential of the combinations because if a driver mutation occurred in *trans*, the effect of the driver mutation might have prevailed.

In the present cohort of patients, somatic mutations in *PIK3CA* were detected in 25% of the patients harboring germline *BRCA1/BRCA2* mutations (two of the eight postmenopausal patients were analyzed for the presence of both gene mutations). This finding may be attributed to the small sample size. In other studies, the frequency of the combination of both mutations appeared to be less than that of individual mutations. In Chinese breast cancer patients, *PIK3CA* somatic mutations were detected in 14% and 43% of the patients harboring germline *BRCA1/BRCA2* mutations (*vs.* wild type carriers), respectively (18). *PIK3CA* somatic mutations were not detected in male patients with breast cancer who harbored *BRCA2* mutations (19).

Although we were not able to identify any associations between the germline *BRCA* and somatic *PIK3CA* mutations because of the small number of patients involved in this study, this is an intriguing situation involving two genes that are treatment targets; therefore, this information may be aggregated in future studies.

The limitations of our study are the small sample size and the sequencing of hotspots (but not all exons of *PIK3CA*), which may have resulted in the underestimation of the mutation frequency. The strengths of this study are the combined analysis of germline *BRCA1/BRCA2* and somatic *PIK3CA* mutations in a group of postmenopausal and young patients with breast cancer.

In conclusion, the present data indicate that *BRCA1/BRCA2* sequencing may be considered for postmenopausal breast cancer patients having a family history of cancer. In addition, although the frequency of *PIK3CA* variants in exons 9 and 20 is high in both elderly and young patients, some of these variants may not be pathogenic in the context of breast cancer.

## AUTHOR CONTRIBUTIONS

Nagy TR conceived the study, enrolled patients, collected clinical data, performed the experiments, analyzed the data, analyzed and interpreted the mutational data, drafted the manuscript, and revised and approved the final version of the manuscript. Maistro S conceived the study, performed the experiments, analyzed the data, analyzed the mutational data, interpreted the data, drafted the manuscript, and revised and approved the final version of the manuscript. Encinas G conceived the study, performed the experiments, analyzed the mutational data, and revised and approved the final version of the manuscript. Katayama MLH performed the experiments, analyzed the data, analyzed the mutational data, interpreted the data, drafted the manuscript, and revised and approved the final version of the manuscript. Pereira GFL analyzed and interpreted the data, drafted the manuscript, and revised and approved the final version of the manuscript. Gaburo-Júnior N and Franco LAM performed the experiments and revised and approved the final version of the manuscript. Gouvêa ACRC, Leite LAS and Diz MPE enrolled the patients, collected the clinical data, and revised and approved the final version of the manuscript. Folgueira MAAK conceived the study, analyzed and interpreted the data, drafted the manuscript, and revised and approved the final version of the manuscript.

## Figures and Tables

**Table 1:** Clinical and pathological features of breast cancer patients according to deleterious *BRCA1* and *BRCA2* mutations.

Features		*BRCA1/BRCA2* mut	*BRCA1/BRCA2* wt
	n=49	n=5	n=44
Age at diagnosis, median (range), years	61 (55-80)	58 (56-80)	62 (55-80)
Age at enrollment, median (range), years	64 (56-87)	60 (58-82)	64.5 (56-87)
Histological grade, n (%)			
I	10	0	10 (100)
II	31	2 (6.5)	29 (93.5)
III	7	3 (42.8)	4 (57.8)
Missing	1	0	1 (100)
Clinical Stage, n (%)			
I	14	0	14 (100)
II	19	1 (5.3)	18 (94.7)
III	10	2 (20)	8 (80)
Missing	6	2 (33.5)	4 (66.5)
Molecular Subtype			
Luminal A	11	0	11 (100)
Luminal B	22	3 (13.7)	19 (86.4)
Luminal	6	0	6 (100)
HER2+	5	0	5 (100)
Triple Negative	5	2 (40)	3 (60)
Affected relatives, n (%)			
First Degree	34	4 (11.8)	30 (82.2)
Second Degree	9	0	9 (100)
Third Degree	4	1 (25)	3 (75)
Negative	2	0	2 (100)
Ancestry until second degree, n (%)			
Brazilian only	13	2 (15.4)	11 (84.6)
European only	9	0	9 (100)
Asian only	5	1 (20)	4 (80)
Brazilian and European	14	1 (7.2)	13 (92.8)
Brazilian and Indigenous	1	0	1 (100)
Brazilian and Australian	1	1 (100)	0
Brazilian and South American	1	0	1 (100)
Brazilian and European and Australian	1	0	1 (100)
Indigenous and European	1	0	1 (100)
European and Unknown	1	0	1 (100)
Indigenous and Unknown	1	0	1 (100)
Unknown	1	0	1 (100)
Region of origin, n (%)			
Southeast	33	2 (6)	31 (94)
Northeast	9	2 (22.2)	7 (77.8)
South	3	0	3 (100)
Abroad	4	1 (25)	3 (75)

**Table 2:** *BRCA1* and *BRCA2* mutations in breast cancer patients: Clinical aspects and molecular description.

ID	HGVS cDNA	HGVS protein	Type	BrCa Age	OvCa Age	Tumor Subtype	HG	CS	Ancestry	FH
*BRCA1*										
29	c.5074+2T>C	-	SS	58	-	TN	2	ND	BRZ	Pos
17	c.5123C>A	p.Ala1708Glu	M	56	-	Lum B	3	III	BRZ/AUS	Pos
47	Exon 1-19 deleted	-	LGR	58	-	TN	3	III	BRZ/EUR	Pos
*BRCA2*										
44	c.2T>G	p.Met1Arg	M	56	-	Lum B	3	II	BRZ	Pos
2	c.5645C>A	p.Ser1882Ter	NS	80	>70	Lum B	2	ND	Asian	Pos

ID: Patient identification; SS: Splice site; M: Missense; LGR: Large genomic rearrangement; NS: Nonsense; Lum: Luminal; HG: Histological grade; CS: Clinical stage; AUS: Australian; FH: Family history of breast, ovarian, pancreatic or prostate cancer; Pos: Positive.

**Table 3:** *In silico* analysis of VUS identified in *BRCA1* and *BRCA2* using mutation function prediction models.

Gene	HDVS cDNA	HGVS protein	SIFT	PolyPhen	Align-GVGD	Provean	Human Splicing Finder	ID
*BRCA1*	c.3305A>G	p.Asn1102Ser	Tolerated	Benign	Class C0	Deleterious	Creation of an exonic ESS site. Potential alteration of splicing.	49
	c.3752G>A	p.Cys1251Tyr	Tolerated	Benign	Class C0	Neutral	Alteration of an exonic ESE site. Potential alteration of splicing.	48
*BRCA2*	c.3371A>G	p.Gln1124Arg	Damaging	Probably Damaging	Class C35	Deleterious	Activation of an exonic cryptic donor site. Potential alteration of splicing.	24
	c.8942A>G	p.Glu2981Gly	Tolerated	Benign	Class C65	Neutral	ND	12

**Table 4:** Clinical and pathological features of breast cancer patients according to their age.

Features	Postmenopausal	Young	
	n=27	n=41	***p***
Age at diagnosis, median (range), years	61 (55-74)	32 (23-35)	
Tumor Subtype			
Luminal A	8 (8)	2 (4.9)	0.04
Luminal B	14 (51.9)	19 (46.3)	
Luminal	2 (7.4)	4 (9.8)	
HER2+	1 (3.7)	5 (12.2)	
Triple Negative	2 (7.4)	10 (24.4)	
Not Determined	0	1 (2.4)	
Clinical Stage, n (%)			
I/II	19 (73.1)	23 (65.7)	0.539
III/IV	7 (26.9)	12 (34.3)	
*BRCA* germline status			
*BRCA1/BRCA2* mut	2 (7.4)	6 (14.6)	0.365
*BRCA1/BRCA2* wt	25 (92.6)	35 (85.4)	
*PIK3CA somatic* status			
*PIK3CA* path mut	10 (37)	7(17.1)	*
*PIK3CA* wt	17 (63)	34 (82.9)	
*Luminal Tumors vs PIK3CA somatic status*	
Luminal *PIK3CA* mut	8 (33.3%)	5 (20%)	0.291
Luminal *PIK3CA* wt	16 (66.7%)	20 (80%)	

Tumor Subtype based on ER, PR, HER2 and Ki67 expression, as described in methods. Missing data were not computed. Pearson's chi-Square. *not tested owing to the small sample size.

## APPENDIX

### ADDITIONAL METHODS

#### NGS

*BRCA1* and *BRCA2* were analyzed for mutations using the Ion AmpliSeq™ *BRCA1* and *BRCA2* panels (Thermo Fisher Scientific). This panel consists of three primer pools (167 amplicons) covering the entire coding region, including 10-20 bp of non-coding sequences flanking the 5’ and 3’ ends of each exon. Library preparation was performed using the Ion AmpliSeq™ Library Kit 2.0 and Ion Xpress™ Barcode Adapter 1-96 kit. DNA amplification was performed using 30 ng of DNA with three primer pools and 5x Ion AmpliSeq™ HiFi Master Mix. The PCR cycle included the following: 2 min at 99°C, followed by 19 cycles of 99°C for 15s and 60°C for 4 min, ending with a hold step at 10°C on a Veriti Thermal Cycler (Thermo Fisher Scientific). Next, the three PCR amplicons were mixed (30 µL), and 20 µL was treated with 2 µL FuPa Reagent to partially digest the primer sequences and phosphorylate the amplicons at 50°C for 10 min, followed by 55°C for 10 min, then 60°C for 20 min, and then held at 10°C. Next, sequencing adaptors (A: conjugated to biotin and P1) and barcodes (consisting of short stretches of index sequences that enable sample multiplexing) were ligated to the amplicons using the Ion Xpress™ Barcode Adapters kit (Thermo Fisher Scientific) for 30 min at 22°C, 5 min at 68°C, and 5 min at 72°C, ending with a hold at 10°C. The adaptor-ligated amplicon (libraries) were purified with 45 μL of the Agencourt^®^ AMPure^®^ XP Reagents (Beckman Coulter) and incubated for 5 min at room temperature(22-25°C). The tube was placed in a magnetic rack such that it was incubated for 2 min or until the solution became clear. After the supernatant was removed carefully and discarded without disturbing the pellet, freshly prepared 70% ethanol (150 μL) was added, and the tube was moved side‐to‐side of the magnet to wash the beads, and then the supernatant was discarded (two rounds of purification were repeated). The tube was placed on the magnet, and the beads were air-dried at room temperature for 5 min. The library was subjected to a second round of amplification using 50 μL of Platinum^®^ PCR SuperMix HiFi and 2 μL of Equalizer™ Primers (added to each bead-pellet); the PCR cycles included 98°C for 2 min, followed by 9 cycles of 98°C for 15s and 64°C for 1 min, ending with a hold at 10°C. Then, 10 μL of Equalizer™ Capture was added to each amplified library, mixed by pipetting, and incubated at room temperature for 5 min. Next, 6 μL of washed Equalizer™ beads was added to each tube containing the captured library, mixed, and incubated at room temperature for 5 min. The tube was then placed in the magnet and incubated for 2 min or until the solution became clear. After the supernatant was removed carefully without disturbing the pellet, the Equalizer™ Wash Buffer (150 μL) was added to each reaction, to wash the beads, the tube was moved side‐to‐side of the magnet, and then the supernatant was removed and discarded (two rounds of purification were repeated). Next, the tube was removed from the magnet and 100 μL of Equalizer™ Elution Buffer was added to each pellet, mixed, and incubated at 32°C in a thermal cycler for 5 min. The tube was placed in the magnet and incubated at room temperature for 5 min or until the solution became clear. The supernatant contained the equalized library at ∼100 pM, and the same amount of the 12 libraries was pooled to perform the emulsion PCR. Next, emulsion PCR was performed using the Ion OneTouch™ System and Ion OneTouch™ 200 Template Kit v2 (Thermo Fisher Scientific). Template-positive Ion Sphere™ Particles (ISPs) were enriched using Dynabeads MyOne™ Streptavidin C1 beads (Invitrogen) and were washed with Ion OneTouch Wash Solution. This process was performed on an Ion OneTouch™ ES System (Thermo Fisher Scientific). The quality of the ISPs was evaluated using a Qubit 2.0 Fluorometer (Invitrogen). The enriched ISPs were sequenced on a 314 v2 Ion Chip (12 samples per chip) using an Ion Torrent Personal Genome Machine (PGM) sequencer system (Thermo Fisher Scientific) using the Ion PGM Sequencing 200 Kit version 2 (Thermo Fisher Scientific). Sequencing was performed using 500 flow runs, which generated approximately 200 bp. The PGM sequencing run outputs were directly loaded to the Torrent Server and stored as ‘.dat’ files. Data analysis comprising annotation of single-nucleotide variants, insertions, deletions, and splice-site alterations was performed using the Ion Reporter™ Server System (Life Technologies). Sequence data were also visually examined and verified using the Integrative Genomics Viewer (IGV). Sequencing generated an average of 302,346 reads per patient, and 96.14% of these regions were mapped to the *BRCA1* and *BRCA2* loci. Amplicons with coverage less than 30x on the Ion Torrent™ platform, as well as pathogenic variants, and new variants were reanalyzed by Sanger sequencing.

#### PCR amplification and Sanger sequencing

The complete coding regions of *BRCA1* (NM_7294.3) and *BRCA2* (NM_000059.3), including 50-100 base pairs (bp) of non-coding sequences flanking the 5’ and 3’ ends of each exon, were amplified by PCR using 33 pairs of primers (for *BRCA1*) (1-2) (Table 1), and 48 pairs of primers (for *BRCA2*) (3) (Table 2). Exons 9 (helical domain) and 20 (kinase domain) of *PIK3CA* (NM_006218.2) were amplified by PCR (Table 3). The PCR products were analyzed by Sanger sequencing in both the forward and reverse directions.

The reaction mixture (total volume, 20 μL) contained AmpliTaq Gold enzyme 250 U (final concentration, 0.04 U/μL) (Applied Biosystems, Foster City, CA, USA), 1× AmpliTaq Gold buffer, 1.5-3.0 mM AmpliTaq Gold magnesium chloride, 0.16 mM deoxynucleotides (Invitrogen, Carlsbad, CA, USA-AM8200), primers (0.4 μM each pair), and 50 ng DNA. PCR was performed on a Veriti^®^ 96-well Thermal Cycler (Applied Biosystems™). The PCR cycle consisted of 1 cycle at 95°C for 10 min, 40 cycles at 94°C for 50 s, 54-66°C for 50s, 72°C for 50s, and 1 cycle at 72°C for 7 min. The *BRCA2* exon 11 fragments were amplified by touchdown PCR, with annealing temperatures decreasing from 63°C to 56°C for fragments corresponding to the beginning of this exon to nucleotide 4526, and annealing temperatures from 68°C to 61°C for fragments corresponding to the end of the exon. PCR products were loaded onto a 1.5% agarose gel, stained with GelRed Nucleic Acid Stain (Biotium, Hayward, CA, USA), and evaluated. PCR products were treated with Illustra™ ExoStar™ 1-Step (GE Healthcare Bio-Sciences, Pittsburgh, PA, USA) and incubated at 37°C for 15 min, followed by incubation at 80°C for 15 min. All PCR products were sequenced in both forward and reverse directions using BigDye^®^ Terminator v3.1 (Applied Biosystems, Foster City, CA, USA-4337456), according to the manufacturer’s instructions. The final product was sequenced on a 3500 Genetic Analyzer (Applied Biosystems™) or ABI 3730 DNA Analyzer (Applied Biosystems™). Sequences obtained were analyzed using Mutation Surveyor DNA Variant Analysis Software (v3.30, SoftGenetics LLC, State College, PA, USA). All pathogenic mutations were confirmed by Sanger sequencing.

#### MLPA

All patients were investigated for large rearrangements, specifically deletions and duplications, using the MLPA commercial kits, *SALSA^®^ MLPA^®^ P002 BRCA1 probemix* (P002-100R) and SALSA^®^ MLPA^®^ P045 *BRCA2*/CHEK2 probemix (P045-100R) (MRC-Holland, Amsterdam, The Netherlands). At first, 80 ng of genomic DNA resuspended in 2.5 μL ultrapure water was denatured for 10 min at 98°C after which 1.5 μL of the probemix mixture was added (0.75 μL of MLPA probe and 0.75 μL of MLPA buffer). The sample DNA and probemix mixture were heated at 95°C for 1 min and then incubated overnight at 60°C (17h). Afterward, ligation was performed using 1.5 μL of ligase buffer A, 1.5 μL of ligase buffer B, 0.5 μL of Ligase-65, and 12.5 μL of water and the reaction was incubated at 54°C for 15 min. The ligase was then inactivated by incubating the reaction at 98°C for 5 min. Amplification was performed by adding 5 μL of Polymerase mix (1 μL of SALSA PCR primers, 0.25 μL of SALSA polymerase, and 3.75 μL of water) and heated at 95°C for 1 min. PCR was carried out for 35 cycles (30s at 95°C, 30s at 60°C, and 60s at 72°C), followed by 20 min at 72°C on a GeneAmp 9700 Thermal Cycler (Applied Biosystems). Then, 1 μL of the PCR product was diluted 1:10 in water, mixed with 0.075 μL GeneScan™ 600 LIZ^®^ dye Size Standard v2.0 (Applied Biosystems-4408399) and 9 μL Hi-Di Formamide (Applied Biosystems-4440753), and incubated at 80°C for 2 min on a GeneAmp 9700 Thermal Cycler (Applied Biosystems). The fragments were analyzed on an Applied Biosystems 3500 Genetic Analyzer (Applied Biosystems™) or ABI 3730 DNA Analyzer (Applied Biosystems™), and analysis was performed using Coffalyser.Net MLPA Analysis Software (MRC-Holland, Amsterdam, Netherlands). To normalize the data, at least three genomic DNA samples obtained from the peripheral blood cells of healthy donors were used as controls in each analysis. Normal values were considered when the ratio was between 0.8 and 1.2.

#### Nomenclature and classification of mutations

Variants were named according to the Human Genome Variation Society (HGVS) nomenclature (4). *BRCA1* and *BRCA2* variants were searched in publicly accessible databases, *i.e.*, BRCA Share™ (5,6), BRCA Exchange (7), BRCA Mutation Database (8), and ClinVar (9); this search was performed between January and June 2020. Gene variants were evaluated using the following *in silico* prediction models: Polymorphism Phenotyping (PolyPhen; v2.2.2) (10), Sorting Intolerant From Tolerant (SIFT; v1.0.3) (11), Align-GVGD (12,13), Protein Variation Effect Analyzer (Provean; v1.1) (14), and Human Splicing Finder (15) to identify variants of unknown clinical significance. Minor allele frequency (MAF) was checked using the 1000 Genomes Project database (16), the Exome Aggregation Consortium (ExAC) (17,18), Global MAF dbSNP (19), Exome Variant Server, NHLBI GO Exome Sequencing Project (ESP) (20), Genome Aggregation Database (gnomAD) (21), Trans-Omics for Precision Medicine (TOPMed) (22), and Brazilian genomic variants (ABraOM) (23).

The variants were then classified according to the recommendations of the American College of Medical Genetics and Genomics in pathogenic, likely pathogenic, benign, likely benign, and variant of uncertain significance (VUS) (24). VUS for BRCA was also checked for co-occurrence with known pathogenic mutations in the same patient. For some variants, we considered that consensus information in ≥2 databases was strong enough to classify them as benign or VUS.

## Appendix

**Additional Table 1:** Clinical and pathological characteristics of breast cancer patients, *BRCA* sequencing, and the multiplex ligation-dependent probe amplification (MLPA) results.

ID	Age Years	HT	HG	ER (%)	PR (%)	HER2	Ki67 (%)	Molecular Subtype	CS	FH	*BRCA*	MLPA
1	71	IDC	2	100	80	Neg.	25	Luminal B	III	Yes	wt	wt
2	80	IDC	2	80	80	Neg.	30	Luminal B	ND	Yes	*BRCA2*	wt
3	66	IDC	1	95	80	Neg.	15	Luminal B	II	Yes	wt	ND
4	61	IDC	2	95	Neg.	Neg.	20	Luminal B	II	Yes	wt	wt
5	61	IDC	2	100	100	Neg.	12	Luminal A	II	Yes	wt	wt
6	74	IDC	1	100	100	Neg.	10	Luminal A	I	Yes	wt	wt
7	66	IDC	2	60	66	Neg.	30	Luminal B	I	Yes	wt	wt
8	61	IDC	2	Pos.	Pos.	Neg.	30	Luminal B	III	Yes	wt	wt
9	73	IDC	2	100	100	Neg.	10	Luminal A	I	Yes	wt	wt
10	57	IDC	ND	90	70	Neg.	10	Luminal A	I	Yes	wt	wt
11	73	IDC	2	95	Neg.	Neg.	15	Luminal B	II	Yes	wt	wt
12	73	IDC	3	100	5	Neg.	ND	Luminal	I	Yes	wt	wt
13	59	IDC	1	Neg.	Neg.	Pos.	18	HER 2	III	Yes	wt	wt
14	62	IDC	2	90	70	Neg.	ND	Luminal	II	Yes	wt	wt
16	60	IDC	1	90	100	Neg.	8	Luminal A	II	Yes	wt	wt
17	56	IDC	3	Pos.	Pos.	Neg.	80	Luminal B	III	Yes	*BRCA1*	wt
18	56	IDC	3	Neg.	Neg.	Neg.	30	TN	I	Yes	wt	wt
19	63	IDC	2	10	Neg.	Neg.	20	Luminal B	I	Yes	wt	wt
20	65	IDC	2	50	Neg.	Neg.	30	Luminal B	II	Yes	wt	wt
21	67	IDC	1	100	100	Neg.	30	Luminal B	II	Yes	wt	wt
22	56	IDC	2	66	1	Neg.	30	Luminal B	II	Yes	wt	wt
23	62	IDC	2	95	1	Neg.	18	Luminal B	I	Yes	wt	wt
24	76	IDC	3	Neg.	Neg.	Pos.	40	HER2	III	Yes	wt	wt
25	60	IDC	2	Neg.	Neg.	Neg.	65	TN	ND	Yes	wt	wt
26	60	IDC	2	Pos.	Neg.	Neg.	30	Luminal B	II	Yes	wt	wt
27	56	IDC	2	Pos.	Pos.	Neg.	30	Luminal B	ND	Yes	wt	wt
28	63	IDC	2	66	66	Neg.	30	Luminal B	ND	Yes	wt	wt
29	58	IDC	2	Neg.	Neg.	Neg.	33	TN	ND	Yes	*BRCA1*	wt
30	62	IDC	1	100	Neg.	Neg.	5	Luminal A	III	Yes	wt	wt
31	60	IDC	2	66	66	Neg.	30	Luminal B	ND	Yes	wt	wt
32	56	IDC	1	90	70	Neg.	10	Luminal A	I	No	wt	wt
33	55	IDC	1	Neg.	Neg.	Neg.	ND	TN	I	No	wt	wt
34	61	IDC	2	95	15	Neg.	20	Luminal B	II	Yes	wt	wt
35	68	IDC	2	66	66	Neg.	10	Luminal A	II	Yes	wt	wt
36	63	IDC	1	90	80	Neg.	10	Luminal A	I	Yes	wt	wt
37	62	IDC	2	95	0,1	Pos.	40	Luminal B	I	Yes	wt	wt
38	59	IDC	2	40	75	Neg.	20	Luminal B	I	Yes	wt	wt
39	63	IDC	2	100	100	Neg.	13	Luminal A	II	Yes	wt	wt
40	63	IDC	2	100	30	Pos.	20	Luminal B	III	Yes	wt	wt
41	62	IDC	2	Pos.	Pos.	Pos.	ND	Luminal	II	Yes	wt	wt
42	77	IDC	3	Neg.	Neg.	Pos.	70	HER2	III	Yes	wt	wt
43	65	IDC	2	>50	>50	Neg.	5-30	Luminal	II	Yes	wt	wt
44	56	IDC	3	90	Neg.	Neg.	30-40	Luminal B	II	Yes	*BRCA2*	wt
45	64	IDC	2	Pos.	Pos.	Neg.	ND	Luminal	II	Yes	wt	wt
46	55	IDC	2	Neg.	Neg.	Pos.	10	HER2	III	Yes	wt	wt
47	58	IDC	3	Neg.	Neg.	Neg.	70	TN	III	Yes	wt	*BRCA1*
48	75	IDC	1	>66	>66	Neg.	<15	Luminal A	I	Yes	wt	ND
49	79	IDC	2	Neg.	Neg.	Pos.	40	HER2	II	Yes	wt	wt
50	80	IDC	2	Pos.	Pos.	Neg.	ND	Luminal	II	Yes	wt	wt

ID: Patient identification; HT: Histological type; HG: Histological grade; ER: Estrogen receptor; PR: Progesterone receptor; CS: Clinical stage; FH: Family history for breast and/or ovarian cancer; ND: Not determined; wt: Wild type; MLPA: Multiplex ligation-dependent probe amplification.

**Additional Table 2:** *BRCA1* variants.

Exon	HGVS Nucleotide	HGVS Protein	Protein	Other names	Type	Localization (GRCh37)	NCBI 1000 Genomes Browser	Global MAF dbSNP	Allele Frequency ExAC	Global MAF 1000 genomes	ESP	gnomAD	TOPMed	ABraOM	SIFT	PolyPhen	Provean	Align-GVGD (Pufferfish)	Human Splicing Finder	BRCA Exchange	BRCA Mutation Database	BRCA Share™	ClinVar	Interpretation	n
1	c.-19-115T>C	-	-	IVS1-115T>C	5'UTR	17: 41276247	rs3765640	0.35363 (G)	-	0,35363	-	0,31688	0,30248	0.304260	-	-	-	-	Mutant type not implemented in HSF yet	Benign / Little Clinical Significance	ND	ND	Benign	Benign	9
2	c.81-14C>T	-	-	IVS2-14C>T	IVS	17: 41267810	rs80358006	-	-	-	0.00069	-	0,00052	0.001642	-	-	-	-	No significant splicing motif alteration detected. This mutation has probably no impact on splicing.	Benign / Little Clinical Significance	ND	1-Neutral	Benign/Likely Benign	Benign/Likely Benign	1
3	c.134+111C>T	-	-	IVS3+111C>T	IVS	17: 41267632	rs8176100	0.00379 (A)	-	0,00379	-	0,00227	0.00128	-	-	-	-	-	Creation of an intronic ESE site. Probably no impact on splicing.	Benign / Little Clinical Significance	ND	1-Neutral	Benign	Benign	1
6	c.301+43A>G	-	-	IVS6+43A>G	IVS	17: 41256841	-	-	-	-	-	-	-	-	-	-	-	-	No significant splicing motif alteration detected. This mutation has probably no impact on splicing.	ND	ND	ND	ND	Uncertain Significance	1
7	c.441+36_441+49delCTTTTCTTTTTTTT	-	-	IVS7+36del14	IVS	17: 41256090_41256103	rs373413425	-	-	-	-	-	-	0.295230	-	-	-	-	No significant splicing motif alteration detected. This mutation has probably no impact on splicing.	Not Yet Reviewed	ND	1-Neutral	Benign	Benign	23
7	c.441+36C>T	-	-	IVS7+36C>T	IVS	17: 41256103	rs45569832	-	-	-	-	0,00009	-	-	-	-	-	-	No significant splicing motif alteration detected. This mutation has probably no impact on splicing.	Not Yet Reviewed	ND	3-UV	Uncertain significance?	Uncertain Significance	2
7	c.441+41C>T	-	-	IVS7+41C>T	IVS	17: 41256098	rs45489593	-	0,00024	-	-	0,00104	-	-	-	-	-	-	No significant splicing motif alteration detected. This mutation has probably no impact on splicing.	Not Yet Reviewed	ND	1-Neutral	Uncertain significance?	Uncertain Significance	1
7	c.442-34C>T	-	-	IVS7-34C>T	IVS	17: 41251931	rs799923	0.09864 (A)	0,17379	0,09864	0,17569	0,17303	0,14802	0.200328	-	-	-	-	No significant splicing motif alteration detected. This mutation has probably no impact on splicing.	Not Yet Reviewed	ND	1-Neutral	Benign	Benign	15
9	c.548-58delT	-	-	c.IVS8-58delT	IVS	17: 41249364	rs8176144	0.33486 (AAAAAA)	-	-	0,27833	0.3005	0,28382	-	-	-	-	-	Alteration of an intronic ESS site. Probably no impact on splicing.	ND	ND	1-Neutral	Benign	Benign	2
9	c.591C>T	p.Cys197=	C197=	710C>T	Syn	17: 41249263	rs1799965	0.00040 (A)	0,00147	0.00040	0,00123	0,00178	0,00076	-	-	-	Neutral	-	Activation of an exonic cryptic donor site. Creation of an exonic ESS site. Potential alteration of splicing.	Benign / Little Clinical Significance	ND	1-Neutral	Benign	Benign	1
11	c.1067A>G	p.Gln356Arg	Q356R	1186A>G	M	17: 41246481	rs1799950	0.02177 (C )	0,04407	0,02177	0,0459	0,05196	0,04129	0.049261	Deleterious (0.01)	Probably Damaging (0.988)	Deleterious	Class C0	Creation of an exonic ESS site. Alteration of an exonic ESE site. Potential alteration of splicing.	Benign / Little Clinical Significance	1-Not pathogenic or of no clinical significance	1-Neutral	Benign	Benign	6
11	c.1971A>G	p.Gln657=	Q657=	2090 A>G	Syn	17: 41245577	rs28897679	0.00639 (C)	0,00217	0,00639	0,00869	0,00605	0,00741	0.005747	-	-	Neutral	-	Creation of an exonic ESS site. Alteration of an exonic ESE site. Potential alteration of splicing.	Benign / Little Clinical Significance	ND	1-Neutral	Benign	Benign	1
11	c.2077G>A	p.Asp693Asn	D693N	2196G>A	M	17: 41245471	rs4986850	0.03355 (T)	0.05681	0,03355	0,05429	0.05451	0.05336	0.056650	Tolerated (0.08)	Benign (0.01)	Neutral	Class C0	Alteration of an exonic ESE site. Potential alteration of splicing.	Benign / Little Clinical Significance	1-Not pathogenic or of no clinical significance	1-Neutral	Benign	Benign	9
11	c.2082C>T	p.Ser694=	S694=	2201C>T	Syn	17: 41245466	rs1799949	0.33646 (A)	0,34827	0,33646	0,29568	0,31633	0,30145	0.302956	-	-	Neutral	-	Activation of an exonic cryptic donor site. Alteration of an exonic ESE site. Potential alteration of splicing.	Benign / Little Clinical Significance	ND	1-Neutral	Benign	Benign	22
11	c.2311T>C	p.Leu771=	L771L	2430T>C	Syn	17: 41245237	rs16940	0.33526 (G)	0,34196	0,33526	0,27764	0,30018	0,28384	0.282430	-	-	Neutral	-	Creation of an exonic ESS site. Potential alteration of splicing.	Benign / Little Clinical Significance	ND	1-Neutral	Benign	Benign	22
11	c.2596C>T	p.Arg866Cys	R866C	2715C>T	M	17: 41244952	rs41286300	-	0,0001	-	-	0,00016	0,00017	-	Deleterious (0)	Probably Damaging (1)	Deleterious	Class C65	Creation of an exonic ESS site. Potential alteration of splicing.	Benign / Little Clinical Significance	1-Not pathogenic or of no clinical significance	1-Neutral	Benign	Benign	1
11	c.2612C>T	p.Pro871Leu	P871L	2731C>T	M	17: 41244936	rs799917	0.45607 (G)	0,41005	0,54393	0,49316	-	0,4893	0.450739	Tolerated (1)	Benign (0)	Neutral	Class C0	Creation of an exonic ESS site. Alteration of an exonic ESE site. Potential alteration of splicing.	Benign / Little Clinical Significance	1-Not pathogenic or of no clinical significance	1-Neutral	Benign	Benign	26
11	c.3113A>G	p.Glu1038Gly	E1038G	3232A>G	M	17: 41244435	rs16941	0.33566 (C)	0,34287	0,33566	0,27903	0,30081	0,28456	0.282430	Tolerated (0.16)	Possibly Damaging (0.606)	Deleterious	Class C0	Creation of an exonic ESS site. Alteration of an exonic ESE site. Potential alteration of splicing.	Benign / Little Clinical Significance	1-Not pathogenic or of no clinical significance	1-Neutral	Benign	Benign	22
11	c.3119G>A	p.Ser1040Asn	S1040N	3238G>A	M	17: 41244429	rs4986852	0.00978 (T)	0.00978 (T)	0,00978	-	0,01109	0,01571	0.035304	Tolerated (0.21)	Possibly Damaging (0.831)	Neutral	Class C0	No significant splicing motif alteration detected. This mutation has probably no impact on splicing.	Benign / Little Clinical Significance	1-Not pathogenic or of no clinical significance	1-Neutral	Benign	Benign	5
11	c.3305A>G	p.Asn1102Ser	N1102S	-	M	17: 41244243	rs80356900	-	0,00002	-	0,00008	0,00001	0,00001	-	Tolerated (0.17)	Benign (0.156)	Deleterious	Class C0	Creation of an exonic ESS site. Potential alteration of splicing.	Not Yet Reviewed	2-Likely not pathogenic or of little clinical significance	3-UV	Uncertain significance?	Uncertain Significance	1
11	c.3548A>G	p.Lys1183Arg	K1183R	3667A>G	M	17: 41244000	rs16942	0.35264 (C)	0,34901	0,35264	0,29525	0,31548	0,30133	0.299672	Tolerated (1)	Benign (0)	Neutral	Class C0	Creation of an exonic ESS site. Alteration of an exonic ESE site. Potential alteration of splicing.	Benign / Little Clinical Significance	1-Not pathogenic or of no clinical significance	1-Neutral	Benign	Benign	22
11	c.3752G>A	p.Cys1251Tyr	C1251Y	-	M	17: 41243796	rs879254079	-	-	-	-	-	-	-	Tolerated (1)	Benign (0.001)	Neutral	Class C0	Alteration of an exonic ESE site. Potential alteration of splicing.	Not Yet Reviewed	ND	ND	Uncertain significance?	Uncertain Significance	1
11	c.4039A>G	p.Arg1347Gly	R1347G	4158A>G	M	17: 41243509	rs28897689	0.00060 (C)	0.00398	0,0006	0,00484	0,00423	0,00481	0.005747	Tolerated (0.09)	Benign (0.071)	Neutral	Class C0	Creation of an exonic ESS site. Alteration of an exonic ESE site. Potential alteration of splicing.	Benign / Little Clinical Significance	1-Not pathogenic or of no clinical significance	1-Neutral	Benign	Benign	2
12	c.4113G>A	p.Gly1371=	G1371=	-	Syn	17: 41243033	rs147448807	0.00160 (T)	0.00050	0,0016	0,00123	0,00156	-	-	-	-	Neutral	-	No significant splicing motif alteration detected. This mutation has probably no impact on splicing.	Likely benign	ND	2-Likely Neutral	Likely Benign	Likely Benign	1
13	c.4308T>C	p.Ser1436=	S1436=	4427T>C	Syn	17: 41234470	rs1060915	0.33626 (G)	0,3431	0,33626	0,27956	0,30142	0,28489	0.283251	-	-	Neutral	-	No significant splicing motif alteration detected. This mutation has probably no impact on splicing.	Benign / Little Clinical Significance	ND	1-Neutral	Benign	Benign	22
15	c.4485-63C>G	-	-	IVS 14-63C>G	IVS	17: 41226601	rs273900734	0.35344 (C)	-	0,35344	-	0,31645	0,30211	0.300493	-	-	-	-	-	Benign / Little Clinical Significance	ND	ND	Benign	Benign	2
16	c.4837A>G	p.Ser1613Gly	S1613G	4956A>G	M	17: 41223094	rs1799966	0.35583 (C)	-	0,35583	0,29817	-	0,30333	0.300987	Tolerated (0.11)	Benign (0.038)	Neutral	Class C0	Alteration of an exonic ESE site. Potential alteration of splicing.	Benign / Little Clinical Significance	1-Not pathogenic or of no clinical significance	1-Neutral	Benign	Benign	22
16	c.4987-92A>G	-	-	IVS16-92A>G	IVS	17: 41219804	rs8176233	0.35463 (C)	-	0,35463	-	0,31276	0,30294	0.300493	-	-	-	-	Alteration of an exonic ESE site. Potential alteration of splicing.-	Benign / Little Clinical Significance	ND	1-Neutral	Benign	Benign	3
16	c.4987-68A>G	-	-	VS16-68A>G	IVS	17: 41219780	rs8176234	0.35463 (C)	-	0,35463	-	0,31483	0,30295	0.301314	-	-	-	-	Alteration of an exonic ESE site. Potential alteration of splicing.	Benign / Little Clinical Significance	ND	1-Neutral	Benign	Benign	3
17	c.5074+2T>C	-	-	IVS17+2T>C	SS	17: 41219623	rs80358089	-	-	-	-	-	-	-	-	-	-	-	Alteration of the WT donor site, most probably affecting splicing	Pathogenic	5-Definitely pathogenic	ND	Pathogenic?	Pathogenic	1
17	c.5075-53C>T	-	-	IVS17-53C>T	IVS	17: 41216021	rs8176258	0.01098 (A)	-	0,01098	0,01708	0,01825	0,01721	0.018062	-	-	-	-	Alteration of an intronic ESS site. Probably no impact on splicing.	Benign / Little Clinical Significance	ND	1-Neutral	Benign	Benign	2
18	c.5123C>A	p.Ala1708Glu	A1708E	5242C>A	M	17: 41215920	rs28897696	-	0,02487	-	0.00023 (T)	-	-	-	Deleterious (0)	Possibly Damaging (0.633)	Neutral	Class C65	Activation of an exonic cryptic acceptor site, with presence of one or more cryptic branch point(s). Creation of an exonic ESS site. Alteration of an exonic ESE site. Potential alteration of splicing.	Pathogenic	5-Definitely pathogenic	5-Causal	Pathogenic?	Pathogenic?	1
18	c.5152+66G>A	-	-	IVS18+66G>A	IVS	17: 41215825	rs3092994	0.34245 (T)	-	0,34245	-	0,31394	0,29599	0.291461	-	-	-	-	No significant splicing motif alteration detected. This mutation has probably no impact on splicing.	Benign / Little Clinical Significance	ND	1-Neutral	Benign	Benign	3
21	c.5304C>T	p.Cys1768=	C1769C	-	M	17: 41203108	rs138493864	0.00060 (A)	0.00002	0,0006	0,00015	0,00013	0,00009	-	-	-	Neutral	-	Creation of an exonic ESS site. Potential alteration of splicing.	Likely benign	ND	ND	Likely Benign	Likely Benign	1

HGVS: Human Genome Variation Society; MAF: Minor allele frequency; ESP: NHLBI Exome Sequencing Project Exome Variant Server; gnomAD: The Genome Aggregation Database; TOPMed: Trans-Omics for Precision Medicine; ABraOM: Brazilian genomic variants; SIFT: Sorting intolerant from tolerant; PolyPhen: Polymorphism Phenotyping; Provean: Protein Variation Effect Analyzer; Align-GVGD: Class C0 (less probable to interfere with protein function), C15, C25, C35, C45, C55, C65 (more probable to interfere with protein function); Syn: Synonymous; IVS: Intervening sequence; M: Missense; SS: splice site; ND, Not determined; n: Number of patients harboring the variant

**Additional Table 3:** *BRCA2* variants.

Exon	HGVS Nucleotide	HGVS Protein	Protein Abbrev	Other names	Type	Localization (GRCh37)	NCBI 1000 Genomes Browser	Global MAF dbSNP	Allele Frequency ExAC	Global MAF 1000 genomes	ESP	gnomAD	TOPMed	ABraOM	SIFT	PolyPhen	Provean	Align-GVGD (Pufferfish)	Human Splicing Finder	BRCA Exchange	BRCA Mutation Database	BRCA Share™	ClinVar	Interpretation	n
2	c.-26G>A	-	-	203G>A	5'UTR	13: 32890572	rs1799943	0.20927 (A)	0,24652	0,20927	0,20883	0,22032	0,21567	0.217570	-	-	-	-	ND	Benign / Little Clinical Significance	ND	ND	Benign	Benign	21
2	c.-15A>C	-	-	214A>C	5'UTR	13: 32890583	rs138705202	0.00080 (C)	0.00022	0,0008	0,00038	0,00064	0,00076	0.002463	-	-	-	-	ND	Not Yet Reviewed	ND	ND	Benign/ Likely Benign	Likely benign	1
2	c.-11C>T	-	-	218C>T	5'UTR	13: 32890587	rs76874770	0.00439 (T)	0,00163	0,00439	0,00584	0,0051	0,00546	0.007389	-	-	-	-	ND	Benign / Little Clinical Significance	ND	ND	Benign	Benign	2
2	c.2T>G	p.Met1Arg	M1R	-	M	13: 32890599	rs80358547	-	0,00001	-	-	0,00001	-	-	Damaging (0.00)	Probably Damaging (0.998)	Deleterious	Class C65	ND	Not Yet Reviewed	5-Definitely pathogenic	5-Causal	Pathogenic?	Pathogenic	1
3	c.125A>G	p.Tyr42Cys	Y42C	353A>G	M	13: 32893271	rs4987046	0.00080 (G)	0,0017	0,0008	0,00246	0,00162	0,00158	0.001642	Tolerate (0.12)	Benign (0.090)	Neutral	Class C0	Activation of an exonic cryptic donor site. Potential alteration of splicing.	Benign / Little Clinical Significance	1-Not pathogenic or of no clinical significance	1-Neutral	Benign	Benign	1
4	c.425+33A>G	-	-	IVS4+33A>C	IVS	13: 32899354	rs200065709	0.00060 (G)	0,00052	0,0006	0,00031	0.00010	0,00029	0.000821	-	-	-	-	No significant splicing motif alteration detected. This mutation has probably no impact on splicing.	Not Yet Reviewed	ND	2-Likely Neutral	Benign/ Likely Benign	Likely benign	1
4	c.425+67A>C	-	-	IVS4+67A>C	IVS	13: 32899388	rs11571610	0.07428 (C)	-	0,07428	-	0,03064	0,03973	0.045156	-	-	-	-	Alteration of an intronic ESS site. Probably no impact on splicing.	Benign / Little Clinical Significance	ND	1-Neutral	Benign	Benign	6
6	c.517-19C>T	-	-	IVS6-19C>T	IVS	13: 32900617	rs11571623	0.00819 (T)	0,00219	0,00819	0,00738	0,00586	0,007	0.003284	-	-	-	-	No significant splicing motif alteration detected. This mutation has probably no impact on splicing.	Benign / Little Clinical Significance	ND	1-Neutral	Benign	Benign	2
8	c.681+56C>T	-	-	IVS8+56C>T	IVS	13: 32903685	rs2126042	0.18590 (T)	-	0,1859	-	0,21627	0,20076	0.184729	-	-	-	-	No significant splicing motif alteration detected. This mutation has probably no impact on splicing.	Not Yet Reviewed	ND	1-Neutral	Benign	Benign	17
10	c.865A>C	p.Asn289His	N289H	1093A>C	M	13: 32906480	rs766173	0.07368 (C)	-	0,07368	0,03055	0,03055	0,03968	0.045156	Damaging (0.003)	Benign (0.278)	Neutral	Class C0	Alteration of an exonic ESE site. Potential alteration of splicing	Benign / Little Clinical Significance	ND	1-Neutral	Benign	Benign	7
10	c.1114A>C	p.His372Asn	H372N	1342 A>C	M	13: 32906729	rs144848	0.24940 (C)	0,27793	0,2494	-	0,22303	0,23657	0.259442	Tolerated (0.35)	Benign (0.00)	Neutral	Class C0	Alteration of an exonic ESE site.	Benign / Little Clinical Significance	1-Not pathogenic or of no clinical significance	1-Neutral	Benign	Benign	19
10	c.1365A>G	p.Ser455=	S455=	1593A>G	Syn	13: 32906980	rs1801439	0.07368 (G)	0,05178	7368	0,03101	0,03048	0,03968	0.045156	-	-	Neutral	-	Alteration of an exonic ESE site. Potential alteration of splicing	ND	ND	1-Neutral	Benign	Benign	7
10	c.1514T>C	p.Ile505Thr	I505T		M	13: 32907129	rs28897708	0.00040 (C)	0,00072	0,0004	0,00077	0,00083	0,00065	0.000821	Tolerated (0.1)	Possibly Damaging (0.651)	Neutral	Class C0	No significant splicing motif alteration detected. This mutation has probably no impact on splicing.	Benign / Little Clinical Significance	1-Not pathogenic or of no clinical significance	1-Neutral	Benign	Benign	1
10	c.1909+92_1909+96del	-	-	IVS10+92del5	IVS	13: 32907615-32907620	rs144549870	0.01577 (TAT)	-	-	-	-	-	0.006568	-	-	-	-	-	ND	ND	ND	Benign	Benign	2
10	c.1910-74T>C	-	-	IVS10-74T>C	IVS	13: 32910328	rs2320236	0.17452 (C)	-	0,17452	-	0,20561	0,20561	rs2320236	-	-	-	-	Creation of an intronic ESE site. Probably no impact on splicing.	Benign / Little Clinical Significance	ND	1-Neutral	Benign	Benign	14
10	c.1910-51G>T	-	-	IVS10-51G>T	IVS	13: 32910351	rs11571651	0.07348 (T)	0,04934	0,07348	0,03056	0,03041	0,03968	0.045977	-	-	-	-	Alteration of an intronic ESS site. Probably no impact on splicing.	Benign / Little Clinical Significance	ND	1-Neutral	Benign	Benign	6
11	c.2229T>C	p.His743=	H743=	2457T>C	Syn	13: 32910721	rs1801499	0.07348 (C)	0,05158	0.07348	0,03129	0,03065	0,03972	0.045156	-	-	Neutral	-	No significant splicing motif alteration detected. This mutation has probably no impact on splicing.	Benign / Little Clinical Significance	ND	1-Neutral	Benign	Benign	7
11	c.2350A>G	p.Met784Val	M784V	2578A>G	M	13: 32910842	rs11571653	0.00359 (G)	0,00031	0,00359	-	0,00023	0,00022	0.002463	Tolerated (1.00)	Benign (0.00)	Neutral	Class C0	Creation of an exonic ESS site. Potential alteration of splicing.	Benign / Little Clinical Significance	3-Uncertain	3-UV	Benign	Benign	1
11	c.2971A>G	p.Asn991Asp	N991D	3199A>G	M	13: 32911463	rs1799944	0.08007 (G)	0,05341	0,08007	0,03725	0,03723	0,0461	0.046798	Tolerated (1.00)	Benign (0.00)	Neutral	Class C0	Alteration of an exonic ESE site. Potential alteration of splicing	Benign / Little Clinical Significance	ND	1-Neutral	Benign	Benign	7
11	c.3264T>C	p.Pro1088=	P1088=	3492T>C	Syn	13: 32911756	rs36060526	0.00679 (C)	0.00238	0,00679	0,00756	0,00762	0,00756	0.006568	-	-	Neutral	-	ND	Benign / Little Clinical Significance	ND	1-Neutral	Benign	Benign	1
11	c.3371A>G	p.Gln1124Arg	Q1124R		M	13: 32911863	rs1555283204	-	-	-	-	-	-	-	Damaging (0.01)	Probably Damaging (1.00)	Deleterious	Class C35	Activation of an exonic cryptic donor site. Potential alteration of splicing.	Not Yet Reviewed	ND	ND	Uncertain significance	Uncertain significance	1
11	c.3396A>G	p.Lys1132=	L1132=	3624A>G	Syn	13: 32911888	rs1801406	0.26677 (G)	0,29449	0,26677	0,27984	0,29762	0,28221	0.283251	-	-	Neutral	-	Alteration of an exonic ESE site. Potential alteration of splicing.	Benign / Little Clinical Significance	ND	1-Neutral	Benign	Benign	23
11	c.3807T>C	p.Val1269=	V1269=	4035T>C	Syn	13: 32912299	rs543304	0.16813 (C)	0,18985	0,16813	0,19111	0,18144	0,18622	0.187192	-	-	Neutral	-	No significant splicing motif alteration detected. This mutation has probably no impact on splicing.	Benign / Little Clinical Significance	ND	1-Neutral	Benign	Benign	23
11	c.4068G>A	p.Leu1356=	L1356=	4296G>A	Syn	13: 32912560	rs28897724	0.00040 (A)	0,00305	0,0004	0,00315	0.00245	0,00312	0.002463	-	-	Neutral	-	ND	Benign / Little Clinical Significance	ND	1-Neutral	Benign	Benign	1
11	c.4090A>C	p.Ile1364Leu	I1364L	4318A>C	M	13: 32912582	rs56248502	0.00439 (C)	0,00172	0,00439	0,00631		0,00577	0.006568	Tolerated (0.76)	Benign (0.001)	Neutral	Class C0	No significant splicing motif alteration detected. This mutation has probably no impact on splicing.	Benign / Little Clinical Significance	ND	1-Neutral	Benign	Benign	2
11	c.4258G>T	p.Asp1420Tyr	D1420Y	4486G>T	M	13: 32912750	rs28897727	0.00399 (T)	0,0068	0,00399	0,00396	0,00794	0,00425	0.001642	Damaging (0.01)	Benign (0.030)	Deleterious	Class C15	ND	Benign / Little Clinical Significance	1-Not pathogenic or of no clinical significance	1-Neutral	Benign	Benign	1
11	c.5418A>G	p.Glu1806=	E1806=	5646A>G	Syn	13: 32913910	rs34351119	0.00679 (G)	0,00233	0,00679	0,0083	0,00764	0,00785	0.006568	-	-	Neutral	-	ND	Benign / Little Clinical Significance	ND	1-Neutral	Benign	Benign	1
11	c.5640T>G	p.Asn1880Lys	N1880K	5868T>G	M	13: 32914132	rs11571657	0.00220 (G)	0,00076	0,0022	0,00315	0,00264	0,00294	0.000821	Damaging (0.05)	Benign (0.167)	Neutral	Class C0	Creation of an exonic ESS site. Potential alteration of splicing.	Benign / Little Clinical Significance	2-Likely not pathogenic or of little clinical significance	2-Likely Neutral	Benign/Likely Benign	Likely benign	1
11	c.5645C>A	p.Ser1882Ter	S1882X	5873C>A	N	13: 32914137	rs80358785	-	0,00002	-	-	0,00002	0,00002	-	-	-	-	-	Alteration of an exonic ESE site. Potential alteration of splicing.	Pathogenic	5-Definitely pathogenic	5-Causal	Pathogenic	Pathogenic	1
11	c.5744C>T	p.Thr1915Met	T1915M	5972C>T	M	13: 32914236	rs4987117	0.00859 (T)	0,02114	0,02114	0,02114	0.00859 (T)	0,01744	0.017241	Tolerated (0.13)	Benign (0.000)	Neutral	Class C0	Creation of an exonic ESS site. Potential alteration of splicing.	Benign / Little Clinical Significance	ND	1-Neutral	Benign	Benign	2
11	c.5768A>C	p.Asp1923Ala	D1923A	5996A>C	M	13: 32914260	rs45491005	0.00020 (C)	-	0,0002	0,0002	0.00054	0.00105	-	Tolerated (0.29)	Benign (0.144)	Deleterious	Class C0	Alteration of an exonic ESE site. Potential alteration of splicing.	Benign / Little Clinical Significance	2-Likely not pathogenic or of little clinical significance	2-Likely Neutral	Benign	Likely benign	1
11	c.6841+53delTATTCAGTAG	-	-	-	IVS	13: 32915384-32915394	-	-	-	-	-	-	-	-	-	-	-	-	Alteration of an intronic ESS site. Probably no impact on splicing.	ND	ND	ND	ND	Uncertain Significance	1
11	c.6841+80delTTAA	-	-	IVS11+80delTTAA	IVS	13: 32915411-32915414	rs11571661	0.26578 (AA)	-	-	-	-	-	0.279605	-	-	-	-	Creation of an intronic ESE site. Probably no impact on splicing.	Benign / Little Clinical Significance	ND	1-Neutral	Benign	Benign	7
14	c.7017G>C	p.Lys2339Asn	K2339N	7245 G>C	M	13: 32929007	rs45574331	0.00679 (C)	0,00228	0,00679	0,00808	0,00764	0,00786	0.006568	Damaging (0.01)	Benign (0.105)	Neutral	Class C0	No significant splicing motif alteration detected. This mutation has probably no impact on splicing.	Benign / Little Clinical Significance	ND	2-Likely Neutral	Benign	Benign	1
14	c.7242A>G	p.Ser2414=	S2114=	7470A>G	Syn	13: 32929232	rs1799955	0.23263 (G)	-	0,23263	0,21136	-	0,22464	0.238095	-	-	Neutral	-	No significant splicing motif alteration detected. This mutation has probably no impact on splicing.	Benign / Little Clinical Significance	ND	1-Neutral	Benign	Benign	19
14	c.7319A>G	p.His2440Arg	H2440R	7547A>G	M	13: 32929309	rs4986860	0.01038 (G)	0,00304	0,01038	0,01054	0,00946	0,00967	0.007389	Tolerated (0.55)	Benign (0.002)	Neutral	Class C0	ND	Benign / Little Clinical Significance	ND	1-Neutral	Benign	Benign	1
14	c.7397T>C	p.Ala2466Val	A2466V	-	M	13: 32929387	rs169547	0.02416 (T)	0,99372	0.97584	0,9777	0,97881	0,98191	0.983580	Tolerated (0.98)	Possibly Damaging (0.793)	Neutral	Class C0	ND	ND	ND	1-Neutral	Benign	Benign	50
14	c.7435+53C>T	-	-	IVS14+53C>T	IVS	13: 32929478	rs11147489	0.07248 (T)	-	0,07248	-	0,0301	0,03924	-	-	-	-	-	Creation of an intronic ESE site.	Benign / Little Clinical Significance	ND	1-Neutral	Benign	Benign	1
15	c.7469T>C	p.Ile2490Thr	I2490T	7697T>C	M	13: 32930598	rs11571707	0.01597 (C)	0,01436	0.01597	0,00161	0,0035	0,00913	0.021346	Tolerated (1.00)	Benign (0.010)	Neutral	Class C45	No significant splicing motif alteration detected. This mutation has probably no impact on splicing.	Benign / Little Clinical Significance	ND	1-Neutral	Benign	Benign	1
17	c.7806-14T>C	-	-	IVS16-14T>C	IVS	13: 32936646	rs9534262	0.46845 (T)	0,52083	0,53155	0,52015	0,54679	0,53151	0.523810	-	-	-	-	No significant splicing motif alteration detected. This mutation has probably no impact on splicing.	Benign / Little Clinical Significance	ND	3-UV	Benign	Likely benign	36
19	c.8460A>C	p.Val2820=	V2820=	8688A>C	Syn	13: 32944667	rs9590940	0.01438 (C)	0,00368	0,01438	0,01299	0,01105	0,01219	0.006568	-	-	Neutral	-	No significant splicing motif alteration detected. This mutation has probably no impact on splicing.	Benign / Little Clinical Significance	ND	1-Neutral	Benign	Benign	2
19	c.8487+47C>T	-	-	IVS19+47C>T	IVS	13: 32944741	rs11571744	0.01617 (T)	-	0,01617	0,01523	-	0,01481	0.006568	-	-	-	-	No significant splicing motif alteration detected. This mutation has probably no impact on splicing.	Benign / Little Clinical Significance	ND	3-UV	Benign	Benign	3
20	c.8632+132dup	-	-	c.IVS20+132insC	IVS	13: 32945368-32945369	rs201392123	0.00899 (CC)	-	0.00899	-	0,00619	0,00754	0.002627	-	-	-	-	ND	Benign / Little Clinical Significance	ND	1-Neutral	Benign	Benign	2
21	c.8755-66T>C	-	-	IVS21-66T>C	IVS	13: 32953388	rs4942486	0.48842 (T)	-	0,51158	-	0,52569	0,51037	0.508210	-	-	-	-	Alteration of an intronic ESS site. Probably no impact on splicing. Creation of an intronic ESE site. Probably no impact on splicing.	Benign / Little Clinical Significance	ND	1-Neutral	Benign	Benign	38
22	c.8851G>A	p.Ala2951Thr	A2951T	9079G>A	M	13: 32953550	rs11571769	0.00998 (A)	0,00785	0.00998	0,00438	0,00363	0,00721	0.013136	Damaging (0.00)	Probably Damaging (1.00)	Neutral	Class C55	No significant splicing motif alteration detected. This mutation has probably no impact on splicing.	Benign / Little Clinical Significance	ND	1-Neutral	Benign	Benign	1
22	c.8942A>G	p.Glu2981Gly	E2981G	9170A>G	M	13: 32953641	rs398122716	-	0,00001	-	-	0,00002	0,00001	-	Tolerated (0.16)	Benign (0.030)	Neutral	Class C65	ND	ND	ND	3-UV	Conflicting interpretations of pathogenicity? Likely benign(1);Uncertain significance(3)	Uncertain significance	1
23	c.9038C>T	p.Thr3013Ile	T3013I	-	M	13: 32953971	rs28897755	-	0,00023	-	0,00046	0,00019	0,0002	-	Tolerated (0.24)	Probably Damaging (0.875)	Neutral	Class C0	ND	Benign / Little Clinical Significance	1-Not pathogenic or of no clinical significance	1-Neutral	Benign	Benign	1
24	c.9257-83G>A	-	-	IVS24-83G>A	IVS	13: 32968743	rs9595456	0.05052 (A)	-	0,05052	-	0,04116	0.04575	0.022989	-	-	-	-	Creation of an intronic ESE site.	Benign / Little Clinical Significance	ND	1-Neutral	Benign	Benign	4
24	c.9257-16T>C	-	-	IVS24-16T>C	IVS	13: 32968810	rs11571818	0.00439 (C)	0,00439	0,00765	0,00592	0,00548	0,00548	0.004926	-	-	-	-	No significant splicing motif alteration detected. This mutation has probably no impact on splicing.	ND	ND	3-UV	Benign	Likely benign	1
27	c.9730G>A	p.Val3244Ile	V3244I	9958 G>A	M	13: 32972380	rs11571831	0.00679 (A)	-	-	0,0083	0,00767	0,00787	-	Tolerated (0.49)	Benign (0.000)	Neutral	Class C0	ND	Benign / Little Clinical Significance	ND	2-Likely Neutral	Benign	Benign	1
27	c.9976A>T	p.Lys3326Ter	K3326X	10204A>T	N	13: 32972626	rs11571833	0.00439 (T)	0,00702	0,00439	0.00646	0,00544	0,00547	0.004926	-	-	-	-	Creation of an exonic ESS site. Potential alteration of splicing. Alteration of an exonic ESE site. Potential alteration of splicing.	Benign / Little Clinical Significance	2-Likely not pathogenic or of little clinical significance	1-Neutral	Benign	Benign	1
27	c.10110G>A	p.Arg3370=	R3370=	-	Syn	13: 32972760	rs28897762	0.00080 (A)	0,00147	0,0008	0,00215	0,0014	0,00131	0.000821	-	-	Neutral	-	Alteration of an exonic ESE site. Potential alteration of splicing.	Benign / Little Clinical Significance	ND	1-Neutral	Benign	Benign	1
27	c.10234A>G	p.Ile3412Val	I3412V	10462 A>G	M	13: 32972884	rs1801426	0.04493 (G)	0,02266	0,04493	0,03729	0,0369	0,04054	0.021346	Tolerrated (0.34)	Benign (0.002)	Neutral	Class C0	Alteration of an exonic ESE site. Potential alteration of splicing.	Benign / Little Clinical Significance	ND	1-Neutral	Benign	Benign	4

HGVS: Human Genome Variation Society; MAF: Minor allele frequency; EXAC: Exome Aggregation Consortium; ESP: NHLBI Exome Sequencing Project Exome Variant Server; gnomAD: The Genome Aggregation Database; TOPMed: Trans-Omics for Precision Medicine; ABraOM: Brazilian genomic variants; SIFT: Sorting Intolerant From Tolerant; PolyPhen: Polymorphism Phenotyping; Provean: Protein Variation Effect Analyzer; Align-GVGD: Class C0 (less probable to interfere with protein function), C15, C25, C35, C45, C55, C65 (more probable to interfere with protein function); Syn: Synonymous; IVS: Intervening sequence; M: Missense; SS: Splice site; ND: Not determined; n: Number of patients bearing the variant

**Additional Table 4:** *In silico* analysis of the alterations in exons 9 and 20 of *PIK3CA* in postmenopausal patients with breast cancer.

Sample ID	Age at diagnosis	Molecular Subtype	Exon	Cdna	Protein	Protein	Mutation Type	ID COSMIC	Polyphen	SIFT	Provean	Align-GVGD
1	71	Luminal B	9	c.1634A>C	p.Glu545Ala	E545A	M	COSM12458	Probably Damaging	Damaging	Deleterious	Class C65
3	66	Luminal B	9	c.1639G>C	p.Glu547Gln	E547Q	M	-	Probably Damaging	Damaging	Neutral	Class C25
8	61	Luminal B	20	c.3075C>T	p.Thr1025=	T1025T	Syn	COSM21451	-	Tolerated	Neutral	-
				c.3140A>T	p.His1047Leu	H1047L	M	COSM776	Benign	Damaging	Neutral	Class C65
9	73	Luminal A	9	c.1629C>T	p.Ile543=	I543I	Syn	COSM5020257	-	Tolerated	Neutral	-
10	57	Luminal A	9	c.1549C>T	p.Leu517=	L517L	Syn	-	-	Tolerated	Neutral	-
17*	56	Luminal B	9	c.1634A>C	p.Glu545Ala	E545A	M	COSM12458	Probably Damaging	Damaging	Deleterious	Class C65
21	67	Luminal B	9	c.1550T>C	p.Leu517Pro	L517P	M	-	Benign	Damaging	Neutral	Class C65
23	62	Luminal B	20	c.3140A>T	p.His1047Leu	H1047L	M	COSM776	Benign	Damaging	Neutral	Class C65
26	60	Luminal B	9	c.1547G>A	p.Arg516Lys	R516K	M	COSM3724545	Benign	Tolerated	Neutral	Class C25
			20	c.3170G>A	p.Trp1057*	W1057X	N	COSM6475611	-	-	-	-
32	56	Luminal A	20	c.3098A>G	Gln1033Arg	Q1033R	M	COSM303947	Possible Damaging	Damaging	Neutral	Class C35
36	63	Luminal A	9	c.1634A>C	p.Glu545Ala	E545A	M	COSM12458	Probably Damaging	Damaging	Deleterious	Class C65
				c.1658_1659delGTinsC	p.Ser553Thrfs*7	S553fs	F	-	-	-	-	-
39	63	Luminal A	9	c.1638G>A	p.Gln546=	Q546Q	Syn	COSM5622324	-	Toleratd	Neutral	-
				c.1664+46G>A	-	-	IVS	-	-	-	-	-
			20	c.3102G>A	p.Glu1034=	E1034E	Syn	-	-	Tolerated	Neutral	-
46	55	HER2	9	c.1634A>C	p.Glu545Ala	E545A	M	COSM12458	Probably Damaging	Damaging	Deleterious	Class C65
				c.1651C>T	p.Leu551=	L551L	Syn	COSM308546	-	Tolerated	Neutral	-
				c.1658_1659delGTinsC	p.Ser553Thrfs*7	S553fs	F	-	-	-	-	-
47*	58	TN	9	c.1622C>T	p.Ser541Phe	S541F	M	COSM6438100	Possible Damaging	Damaging	Deleterious	Class C65
			20	c.3110A>T	p.Glu1037Val	E1037V	M	-	Benign	Damaging	Deleterious	Class C65

HGVS: Human Genome Variation Society; SIFT: Sorting Intolerant From Tolerant; PolyPhen: Polymorphism Phenotyping; Provean: Protein Variation Effect Analyzer; Align-GVGD: Class C0 (less probable to interfere with protein function), C15, C25, C35, C45, C55, C65 (more probable to interfere with protein function); Syn: Synonymous; IVS: Intervening Sequence; M: Missense; N: Nonsense. *Patients also harboring pathogenic germline mutations in *BRCA1*.

**Additional Table 5:** *In silico* analysis of the alterations in exons 9 and 20 of *PIK3CA*in young patients with breast cancer.

Sample ID	Age at diagnosis	Molecular Subtype	Exon	cDNA	Protein	p.Asn1044Asp	Mutation Type	ID COSMIC	Polyphen	SIFT	Provean	Align-GVGD
452	34	Luminal B	20	c.3130A>G	p.Asn1044Asp	N1044D	M	COSM27134	Probably Damaging	Tolerated	Neutral	Class C15
454	34	Luminal	20	c.3146G>A	p.Gly1049Asp	G1049D	M	COSM308548	Probably Damaging	Tolerated	Neutral	Class C65
455	28	Luminal B	9	c.1558G>A	p.Asp520Asn	D520N	M	COSM29096	Benign	Tolerated	Neutral	Class C15
457	33	Luminal B	9	c.1615C>T	p.Pro539Ser	P539S	M	COSM249880	Probably Damaging	Tolerated	Deleterious	Class C65
				c.1664G>A	p.Arg555Lys	R555K	M	COSM1716158	Probably Damaging	Damaging	Deleterious	Class C25
468	33	Luminal A	9	c.1656G>A	p.Trp552*	W552X	N	COSM37025	-	-	-	-
477	27	TN	9	c.1634A>C	p.Glu545Ala	E545A	M	COSM12458	Probably Damaging	Damaging	Deleterious	Class C65
478	29	HER2	20	c.3165G>A	p.Met1055Ile	M1055I	M	COSM9146166	Benign	Tolerated	Neutral	Class C0
				c.3201G>A	p. Leu1067=	L1067L	Syn	-	-	Tolerated	Neutral	-
480	31	TN	9	c.1634A>C	p.Glu545Ala	E545A	M	COSM12458	Probably Damaging	Damaging	Deleterious	Class C65
			20	c.3203A>C	p.Asn1068Thr	N1068T	M	-	Probably Damaging	Damaging	Neutral	Class C55
483	35	Luminal B	9	c.1634A>C	p.Glu545Ala	E545A	M	COSM12458	Probably Damaging	Damaging	Deleterious	Class C65
484	35	Luminal B	9	c.1593C>A	p.Leu531=	L531L	Syn	-	-	Tolerated	Neutral	-
503	35	HER2	20	c.3150C>T	p.Gly1050=	G1050G	Syn	-	-	Tolerated	Neutral	-
518	31	Luminal B	9	c.1615C>T	p.Pro539Ser	P539S	M	COSM249880	Probably Damaging	Tolerated	Deleterious	Class C65

HGVS: Human Genome Variation Society; SIFT: Sorting Intolerant From Tolerant; PolyPhen: Polymorphism Phenotyping; Provean: Protein Variation Effect Analyzer; Align-GVGD: Class C0 (less probable to interfere with protein function), C15, C25, C35, C45, C55, C65 (more probable to interfere with protein function); Syn: Synonymous; M: Missense; N: Nonsense.
